# Notes and News

**Published:** 1889-08-03

**Authors:** 


### Notes and News.

Collected and Communicated.

The Court of Common Council of the City have voted fifty guineas to the Children's Country Holidays Fund.

A unique feature at the Central London Throat and Ear Hospital, Gray's-inn-road, has been the educational treatment of sufferers from defects of speech, which has from the first been carried out by Mr. William Van Praagh. At the recent annual meeting the committee passed a cordial vote of thanks to this gentleman for his arduous and gratuitous labours in this department, and, as a slight recognition of his unwearying kindness, presented him with a silver salver suitably inscribed.

At the annual demonstration of Stockton Friendly Societies on behalf of the hospital, Sir Horace Davey said the most satisfactory feature in the progress of the hospital had been that the average cost of the accommodation of the patients had been largely reduced. In 1873 the average cost per day of each person was 4s. 6d., but it was now under 2s., a mere fraction over 1s. 11d. per day per person. The Chairman, in responding, stated that the collection in the streets had amounted to £29 8s. 9d., and the collection in the theatre £24 16s. 7½d., or altogether £54 5s. 4½d., a sum considerably in excess of last year's.

A Home and Health Exhibition was open last Friday and Saturday at Kensington Town Hall. In the evening, entertainments were given, in which Dr. Hare, Professor Henslow, and others took part. Promoted by the Bread and Food Reform League, whose object is to show how greatly health and economy depend upon the use of suitable food, it showed samples of different food, and of arrangements for preparing them, among the latter being a model dairy. Wheat-meal bread, which the League strongly advocates, occupied a prominent place; but to many visitors the most interesting portion of the exhibition was the potter working at his wheel.

Birmingham Hospital Saturday Fund has been divided as follows:—To the General Hospital, £2,680 (increase £193); Queen's Hospital, £1,345 (increase £61); General Dispensary, £919 (increase £88); Eye Hospital, £691 (increase £36); the Sanatorium, £322 (increase £29); Homœopathic Hospital, £251 (increase £34); and about £1,300 to the lesser charities, including £524 to the Jaffray Suburban Hospital; altogether, £8,300, leaving £452 to cover expenditure, and a balance of £48 to be carried forward. The Birmingham collection exceeds those of Manchester, Liverpool, and Sheffield combined, and roughly speaking the working-men of Birmingham supply a third of the income of their hospitals, a most creditable state of affairs.

The report on Dublin hospitals is out, and reports most favourably of Steeven's Hospital; the Lock Hospital is said to be very gloomy. Cork-street Fever Hospital, they say, continues to be worked efficiently, and its sanitary condition is satisfactory. The wards are well ventilated and scrupulously clean. The infectious diseases are treated in separate wards, and there are special nurses for each. They also report very favourably of the House of Industry Hospital. The Rotunda Hospital is stated to be conducted with efficiency, and the most rigid attention is given to details as regards cleanliness and order. The same observations in substance are made with regard to the Coombe Hospital. With respect to St. Mark's Ophthalmic Hospital they fear that the structural defects and over-crowding of some of the wards mar its work to a great extent. Of the Hospital for Incurables they speak in high terms, and mark amongst its improvements the provision for extinguishing fires. We wonder if the Board knew that the nurses at the Rotunda sleep in the wards with the patients?

On Monday last Lord Sandhurst brought a petition before the House of Lords. It is signed by a large number of medical men, and by representatives of various charitable bodies, and prays for an enquiry into the "financial and general management and common organisation" of the institutions, endowed, voluntary, and municipal, which give aid to the sick poor within the Metropolis. The movement of which this action is the outcome has been set on foot by the Charity Organisation Society. First among the formidable list of defects put forward by the society's petition is the allegation that the out-patient departments of the hospitals are largely made use of by persons suffering from slight ailments, and that thereby a constant hindrance is occasioned to medical instruction. Then it is asserted that there is no proper distribution of work between voluntary hospitals and dispensaries and the poor-law establishments for performing the same functions, and that, accordingly, "the former deal with cases which might more properly be left to the Poor Law, and the latter with cases which, from their medical interest and special requirements, or from the character and circumstances of the patient, might more properly be treated in charitable institutions." The multiplication of gratuitous and part-paying institutions and the absence of organisation tend, besides, to deprive those medical men whose practice lies among the poor of all chance of making a livelihood. Last of all, it is pointed out that there is no regular co-operation or agreement between the different establishments as to the transference of cases from dispensary to hospital, and *vice versâ*, according as the nature of the patient's ailments may make it desirable, and that no uniform plan of keeping and publishing accounts is now in existence. Many of these points have been constantly urged in this paper, and we are glad that they have been taken up by Lord Sandhurst, whose action should cause great results.

The following vacancies are announced this week, the amount of the salary in each case being given after the name of the institution :

**Resident Medical Officers.**

Somerset and Bath Lunatic Asylum. Junior Assistant Medical Officer.—£100, board, &c.
Wisbech Hospital. House Surgeon.—£130, board, &c.
Ripon Cottage Hospital. House Surgeon and Dispenser.—£70, board, &c.

**Non-Resident Medical Officers.**

City of London Hospital, Victoria Park. Physician.
London Temperance Hospital. Registrar and Chloroformist.—£50.
Hove. Medical Officer of Health.—£100.
West. Gen. Dispensary, Marylebone. Hon. Dental Surgeon.
Towcester Union. Medical Officer.—£100.

Dr. Robertson, chairman of Devonshire Hospital and Buxton Bath Charity, has issued his half-yearly address: "During the half-year, ending June 30, 1,152 in-patients were admitted, and of this number 862 were discharged as improved, only 26 as no better, 9 were discharged at their own request, 2 were found to be unfit cases for the hospital, 4 were found to be infested with vermin, 2 had to be sent away for misconduct, 2 left without having reported themselves, 3 were sent away on account of drunkenness, the unusual number of 5 had died in the hospital, and 237 remained on the books at the end of the half-year. It will be seen that of the total 1,152 in-patients received during the half-year, 1,002 were of rheumatic or gouty character, and that 441 were of what has been called rheumatism proper, 421 of rheumatic arthritis, a probably unusual proportion of this inveterate form of rheumatism, 10 of rheumatic synovitis, 22 of gout, 3 of gout complicated, or occasioned by lead poisoning, 31 of lumbago, and 72 of sciatica. It will be noticed, that no fewer than 121 of these cases were complicated with heart-disease, or more than one in nine of the total rheumatic cases admitted.

The laying of the stone of the New Branch of the Dreadnought Seamen's Hospital by Prince George of Wales was a pretty sight, and ought to have been reported before in these pages. His Royal Highness, who was attended by Captain Stevenson, R.N., journeyed by special train from Fenchurch-street Station, and reached the Central Station, Royal Albert Dock, shortly before four o'clock. Here he was received by Mr. Frederick Cleeve, R.N., and other members of the committee of management of the Seamen's Hospital, and by a guard of honour of the 15th Middlesex Rifle Volunteers. Entering a carriage, the Royal party drove to the site of the new hospital, where a spacious marquee had been erected, the entire route from the station, extending for about half-a-mile being lined by the boys of the Naval School and sailors of all nations in their various uniforms, the Eastern costumes of the Lascars being specially effective. Unluckily, a smart shower of rain, which even penetrated the marquee, occurred about four o'clock. Prince George said in his speech: "I am well aware, from the visits which I have paid to distant parts of the world as a member of the service to which I have honour to belong, of the hardships and privations to which seamen—and especially those of the mercantile marine—are frequently exposed, and I feel sure, therefore, that the proposed extension of your philanthropic work will prove of the greatest utility." The new branch hospital will accommodate twelve patients, together with the medical and nursing staff, and there will be an out-patient department. The whole arrangements reflected great credit on the chairman, Mr. Cleeve, C.B., and the secretary, Mr. Michelli, who are to be heartily congratulated upon a genuine and deserved success.

Mr. Mowbray is architect of the new Children's Hospital at Eastbourne, which is to cost £10,000. The building will be red brick, with a little stonework to relieve the monotony; it will be heated by steam, and well ventilated. There are to be covered playgrounds for wet weather; lifts for patients and goods; bedrooms adjoining day-rooms for cripples; and conveniences of all kinds to render the working of the hospital as efficient as the latest scientific and mechanical discoveries will permit.

The Hospital for Epilepsy and Paralysis issues a very nicely got-up report, and, what is more, the accounts are neatly balanced. We specially admire the clearness of the list of annual subscriptions and donations. The hospital specially appeals for more subscribers of a guinea. The annual deficit exceeds £1,000, and can only be met in this way, or by making costly appeals. In-patients 108, out-patients 8,932, were treated last year. Mr. Howgrave Graham, the secretary, has laboured assiduously to make this institution efficient, and its present prosperous state is largely due to his zeal and ability.

On Monday last a select party of visitors went from London to St. Margarets to visit the Morley House Seaside Convalescent Home for Working Men. The party included the Earl of Aberdeen, the Lady Mayoress, Mr. Sheriff Newton, and others. The whole village and the home were decorated in honour of the occasion. It is an exceedingly pleasant little home of rest for thirty-six convalescents. Its lawns and flower-beds and shrubs and trees and kitchen garden, and its salt sea breezes all combine to afford just the sort of retreat for working-men slowly feeling their way back to complete health and strength. They generally stay here for three weeks, paying five shillings a week. The chief point about the hospital is the economy with which it is managed: £100 a year covers every expense, but, unluckily, the home is in debt. Lord Aberdeen is the president.

The report of the Manchester Infirmary Board just issued states that during the year now terminated 43,985 persons have received assistance at the Royal Infirmary and its allied institutions at Cheadle and Monsall. Of this number 4,504 were in-patients, 1,378 were sent to the Convalescent Hospital at Cheadle, 1,960 were admitted at the Fever Hospital at Monsall, and 36,143 were treated as out and home patients. Further benefits were received by 271 patients who were sent to the hospitals at Southport or Buxton through the kindness of the governors of the Cotton Districts Convalescent Fund. In the last report attention was drawn to the fact that the number of out and home patients had, year by year, rapidly increased. During the last five years the numbers have been 23,263, 27,295, 31,761, 33,288, and 36,143 respectively. This increase has taken place, although strict investigations are made, after the first visit, into the circumstances of every applicant; some are refused further relief, and those who are retained on the books are specially invited to contribute to the cost of their treatment. The income of the infirmary and convalescent hospital for the past and previous year has been as follows:—1888-9, Royal Infirmary, £20,046; Convalescent Hospital, £2,285; 1887-8, Infirmary, £20,875; hospital, £2,659, showing a reduction of £1,203. The expenditure at the Royal Infirmary during the last 12 months has been £16,324, the lowest amount since the year 1866-7. At the Convalescent Hospital at Cheadle the expenditure has been £5,190, or £45 more than last year. At the Monsall Fever Hospital the expenditure has been £8,873, or £266 more than in the year ending June 24, 1888.

